# Influenza Vaccination among Pregnant Women: Patient Beliefs and Medical Provider Practices

**DOI:** 10.1155/2016/3281975

**Published:** 2016-07-31

**Authors:** Lauren M. Stark, Michael L. Power, Mark Turrentine, Renee Samelson, Maryam M. Siddiqui, Michael J. Paglia, Emmie R. Strassberg, Elizabeth Kelly, Katie L. Murtough, Jay Schulkin

**Affiliations:** ^1^Research Department, American College of Obstetricians and Gynecologists, 409 12th Street SW, Washington, DC 20024, USA; ^2^Department of Obstetrics & Gynecology, Kelsey-Seybold Clinic, 1111 Augusta Drive, Houston, TX 77057, USA; ^3^Division of Maternal Fetal Medicine, Department of Obstetrics & Gynecology, Albany Medical Center, 391 Myrtle Avenue, Suite 2, Albany, NY 12208, USA; ^4^The University of Chicago Medicine, 5841 S. Maryland Avenue, MC 2050, Chicago, IL 60637, USA; ^5^Department of Maternal-Fetal Medicine, Geisinger Health System, 100 N Academy Avenue, Danville, PA 17822, USA; ^6^Department of Obstetrics & Gynecology, Albany Medical Center, 391 Myrtle Avenue, 2nd floor, MC 74, Albany, NY 12208, USA; ^7^American College of Obstetricians and Gynecologists, 409 12th Street SW, Washington, DC 20024, USA

## Abstract

ACOG's research department recruited four medical centers to participate in a study on the attitudes and practices of medical providers and pregnant patients regarding influenza vaccination. Medical providers and patients were given voluntary surveys and medical record data was collected over two flu seasons, from 2013 to 2015. Discrepancies between self-reports of medical providers and patients and medical records were observed. Nearly 80% of patients self-reported accepting the influenza vaccine, but medical record data only reported 36% of patients accepting the vaccine. Similarly, all medical providers reported giving recommendations for the vaccine, but only 85% of patients reported receiving a recommendation. Age, education, a medical provider's recommendation, and educational materials were found to positively influence patient beliefs about the influenza vaccine. Accepting the vaccine was influenced by a patient's previous actions, beliefs, and a medical provider's recommendation. Patients who reported previously not accepting the vaccine and had negative feelings towards the vaccine but accepted it while pregnant reported concern for the health and safety of their baby. Future research should focus on groups that may be less likely to accept the vaccine and ways to dispel negative myths. Medical provider should continue to strongly recommend the vaccine and provide educational materials.

## 1. Introduction

Influenza vaccination is recommended during pregnancy to prevent harm to both mothers and fetuses. Pregnant women are at an increased risk of developing serious illness from the flu due to changes within their immune system; as well they are at risk for complications such as premature birth if they develop the flu [1]. While many anti-immunization groups have raised concerns over ill effects of vaccinations, no scientific evidence has supported their claims [2, 3]. Additionally, accepting the vaccination is associated with lower rates of influenza diagnosis, which, if contracted, is associated with higher rates of fetal mortality [4]. In 2014, the American College of Obstetricians and Gynecologists (ACOG) reaffirmed their committee opinion recommending that all pregnant women should accept the influenza vaccination, unless there are valid medical reasons such as allergic reactions. Despite the preventive benefits from the influenza vaccine, during the 2014-2015 flu season, only 50% of pregnant women accepted the influenza vaccine [2]. This is well below the 80% goal set by Healthy People 2020, a national government program that has established benchmarks to improve health outcomes of Americans [5]. Studies suggest that pregnant women are concerned about contracting influenza from the vaccine and about harming their baby, which decreases their likelihood of accepting the vaccine while pregnant [6, 7]. In a review of barriers associated with immunization in pregnancy, however, Shavell et al. noted multiple studies which found that concern for their baby can increase vaccination rates among pregnant women and that a physician's recommendation has a strong impact on vaccination rates [7]. In 2013, ACOG launched a study on influenza vaccination among pregnant women through the Expanded Collaborative Ambulatory Research Network (ECARN). The purpose of this study was to understand the attitudes and practices of patients and medical providers regarding the influenza vaccination, as well as identifying potential barriers that exist in vaccine acceptance among pregnant women.

## 2. Materials and Methods

Since 1995, the Collaborative Ambulatory Research Network (CARN) conducted by ACOG has surveyed obstetricians and gynecologists (OB/GYNs) on their knowledge, attitudes, and beliefs regarding a number of obstetric and gynecological issues to provide up-to-date information for improving educational materials and ACOG guidelines. One of the main goals of CARN is to improve patient health by understanding the resources and guidelines OB/GYNs need, as well as the barriers they perceive to providing quality healthcare. ECARN expands on this effort by providing access to patients and their medical records. In this way, provider perspectives can be assessed in conjunction with patient perceptions and medical record data.

ECARN was created to help bridge the data gap between providers, patients, and records. Medical centers and private OB/GYN offices are enlisted to take part in studies that collect data from medical providers and patient self-reports, as well as medical record data. Communication between medical providers and patients is imperfect, and both medical provider and patients surveys are subject to biased responses based on social desirability and recall error. By comparing the two perspectives and including medical record data, discrepancies can give insight into where additional resources or education is needed.

Four sites were recruited for the ECARN study, which varied in location, size, and demographics (see Table 1): Kelsey-Seybold Clinic (Texas), the Women's Health Center of Albany Medicine (New York), the University of Chicago Medicine (Illinois), and Geisinger Health System (Pennsylvania). As an expansion of CARN, ECARN sites were recruited from the CARN member database from those who had expressed interest in conducting patient-centered research. Data collection was overseen by a lead medical provider at each site, who was responsible for collecting materials, shipping them to ACOG, and serving as a lead for answering any questions by medical staff or patients. Individual sites received approval from a local IRB, and ACOG research staff created and provided all sites with the appropriate materials for data collection, including patient and medical provider surveys, patient information sheets, data collection procedures for staff, site surveys, and return mailing materials. Data was collected during the 2013-2014 flu season, September 2013 to April 2014, and during the 2014-2015 flu season, September 2014 to April 2015.

Patients were recruited by medical staff who were knowledgeable of the study and provided with an information sheet describing voluntary consent to participate. All pregnant patients were provided a questionnaire regarding their opinions and practices of the influenza vaccination and medical providers filled out a data sheet at the time of their visit. Each patient's medical record was marked to show they received the survey, but no identifiable information was collected with the survey or data sheet. All medical providers were similarly given a questionnaire to fill out at the beginning of data collection. In addition to OB/GYNs, one site also included non-MD obstetric providers (e.g., nurse midwives); however, given the small number (6), all medical providers were combined together for analysis. Two sites collected prospective medical record data on forms provided by ACOG which recorded basic information such as status of receiving the influenza vaccine and delivery outcomes. The two other sites conducted a retrospective data pull from their electronic medical records (EMR) after their survey collection period had ended. These results are discussed individually and combined as “medical record data” below.

All surveys were sealed in envelopes and collected by individual sites. Surveys and data sheets were sent to ACOG for analysis after the end of data collection. No identifying information was collected. Results were analyzed aggregately, by site, and combined. The majority of data was entered by hand by ACOG staff into Microsoft Excel 2013^©^, with the exception of two sites who provided an electronic format of their medical records. Data was analyzed using IBM SPSS Statistics 20.0, IBM Corp.^©^, Armonk, NY.

All responses indicating the patient had already accepted or had the intention to accept the influenza vaccine during pregnancy were grouped together and are referred to as accepted in this paper. These groups were combined due to variance in survey participation dates. Some patients received the survey at the beginning of the flu season; thus it was unlikely that they had accepted the vaccine already, and others received the survey at the end of the flu season. This is further discussed as a limitation below. A binary logistic regression was used to determine the significance of factors for accepting the influenza vaccination (no or yes). Independent variables included previous influenza vaccination practices, site, age, education, race, receiving a medical provider's recommendation, receiving educational materials, and patient belief scores. Similarly, a multinomial logistic regression was used to analyze factors influencing patient beliefs. Patient beliefs score ranged from 4 to 20, adding the score of 4 different questions pertaining to patient attitudes on their own and their baby's health and safety regarding the influenza vaccine. Each question asked patients to rate their beliefs on a 5-point scale, from “strongly disagree” (1) to “strongly agree” (5). Patient belief scores were grouped into 3 categories (4–9, 10–15, and 16–20) for the multinomial analysis. Similar to the binary logistic model, independent variables included previous influenza vaccination practices, site, age, education, race, receiving a medical provider's recommendation, and receiving educational materials.

Patients were compared based on their previous influenza vaccination practices. Four groups were created to compare patient belief scores. Group A was comprised of patients who previously did not and continued not to get the vaccine. Group B was comprised of patients who previously accepted the vaccine but did not while pregnant. Group C was comprised of patients who previously did not but decided to get the vaccine while pregnant. Finally, Group D was comprised of patients who previously accepted the vaccine and continued to accept the vaccine when pregnant. An exploratory analysis of differences between the groups was conducted using Pearson chi-square tests.

## 3. Results 

A total of 76 providers and 984 patients responded to the surveys (see Table 1). Providers were asked about their attitudes and practices towards administration of the influenza vaccine, and patients were asked about their beliefs and practices towards getting the influenza vaccine. Medical records data served as a backup to the recall issues that come from surveys to show how many patients actually accepted the vaccination. Provider and patient perspectives, along with medical record data, are analyzed and compared below.

### 3.1. Medical Providers Self-Reports

The 76 medical providers included obstetrician-gynecologists attending physicians and residents, nurse practitioners, physician assistants, and nurse midwives and varied demographically across the four sites (Table 1). The only statistically significant difference was that male providers were more likely to be older, and, similarly, in clinical practice for more years than female providers (*p* < .01). Nearly all providers responded that primary or preventive care was an important part of their practice; 48.7% answered “very important,” and 48.7% answered “important.”

Providers were asked about their attitudes regarding safety of the influenza vaccine (Table 2). Neither site nor age was a significant factor in determining beliefs. Nearly one-third of medical providers (31.5%) were concerned or very concerned about safety of administration of influenza vaccine in the 1st trimester. Only in site 4 did a demographic variable prove statistically significant; female providers were more concerned than male providers (*p* < .01). All providers were concerned with the health of the mother and fetus if the mother contracted the flu during pregnancy (82.9% very concerned, 15.8% concerned, and 1.3% slightly concerned).

All providers recommended the influenza vaccine to their pregnant patients (Table 3). The majority (90.7%) recommended it during any trimester, and the remaining 9.3% recommended it any time after the 1st trimester. Sites varied in their responses; all of Site 1 providers recommended the vaccination during any trimester, whereas the remaining sites differed. However, this discrepancy was not statistically significant. Similarly, practice site was not statistically significant for providing educational materials, despite variation among responses (Table 3). Overall, 53.9% of providers reported they always provided educational materials, 27.6% sometimes provided educational materials, and 15.8% never provided educational materials. Within sites, gender was a significant factor for Sites 2 and 3. In Site 2, women were more likely to never or only sometimes provide educational materials than men (50% versus 22.2%; *p* = .047); however the opposite was found in Site 3 (100% of men never provided educational materials versus only 20% of women; *p* < .01).

### 3.2. Patient Self-Reports

Overall, 77.9% of patients stated that they had already (71.1%) or were planning on (6.8%) accepting the influenza vaccine while pregnant. Individual sites ranged from 69.7% to 87.1% acceptance rates. The majority of patients who listed where they accepted the vaccination received it from their respective hospital group. Binary logistic regression was used to determine the significance of factors for accepting the influenza vaccination within individual sites and aggregated (see Supplementary Table  6 in Supplementary Material available online at http://dx.doi.org/10.1155/2016/3281975). Within individual sites, the only significant variable was accepting the influenza vaccine in previous years. At Site 1, sometimes accepting the influenza vaccination increased the odds ratio by 3.804 (*p* = .012) and usually or always by an odds ratio of 27.296 (*p* < .01). At Site 2, sometimes accepting the influenza vaccination increased the odds ratio by 3.288 (*p* < .01) and usually or always by an odds ratio of 24.809 (*p* < .01). At Site 3, usually or always accepting the influenza vaccination increased the odds ratio by 18.308 (*p* < .01). At Site 4, sometimes accepting the influenza vaccination increased the odds ratio by 3.667 (*p* < .01) and usually or always by an odds ratio of 35.648 (*p* < .01).

Aggregated, the patient's individual beliefs and practices made a difference in vaccination acceptance. The binary logistic regression produced a Nagelkerke *R* square of .653 and the Hosmer and Lemeshow test produced a significance level of .683, indicating that the model is good fit. Among the covariates, for every 1-point increase in a patient's influenza vaccine belief score, the odds ratio of accepting the vaccine was increased by 1.708 (*p* < .01). Additionally, accepting the influenza vaccine usually or always in previous years increased the probability of accepting the influenza vaccine while pregnant, by an odds ratio of 4.867 (*p* < .01). Finally, a medical provider's recommendation also increased the odds ratio of accepting the vaccine by 2.603 (*p* < .01). Medical center site, education level, age, receiving educational materials, and race were not statistically significant.

Overall, patients had positive beliefs about the vaccination, which, as stated above, was a significant factor in accepting the influenza vaccination. Nearly half (49.5%) scored between 16 and 20, and another 40.8% scored between 10 and 15. Only 9.7% of patients scored between 4 and 9. Nearly all patients who scored above 16 accepted the influenza vaccination (98.3%), and most patients with a score of 4–9 declined the vaccine (84%). Patients who scored in the middle (10–15) produced the highest variation; 68.4% accepted the vaccination, and 31.6% did not accept the vaccination.

A multinomial logistic regression determined a number of factors which were statistically significant in the patient reporting positive beliefs about the influenza vaccine (see Supplementary Table  7). The likelihood ratio chi-square was 106.752 (*p* < .01) and the Nagelkerke *R* square was .125. Only three independent variables were found to be statistically significant. Patient age was statistically significant to score 10–15 in comparison to the scoring in the lowest group, increasing the odds ratio by 1.056 (*p* = .027) for every 1-year increase. Patient age was also statistically significant in scoring 16–20; for every 1-year increase, the odds ratio increased by 1.095 (*p* < .01). In addition, having a high school degree decreased the probability of scoring 16–20 by 92.1% (odds ratio of .079; *p* = .015). Finally, receiving educational materials increased the odds ratio of scoring 16–20 by 2.287 (*p* < .01).

As stated above, the patient belief score had a strong effect on the likelihood of accepting the influenza vaccine while pregnant. To further explore this, we compared four groups of patients based on their past immunization history and acceptance of vaccination while pregnant (e.g., previously did not accept the vaccine but accepted the vaccine while pregnant; see Tables 4 and 5). Most interesting are groups B and C, patients who changed from their previous practice patterns. The majority of Group C answered “don't know” for believing people should get the influenza vaccine, and the rest disagreed more than agreed, however, for the remainder of the questions that indicated the importance of vaccine while pregnant and for the safety of their baby. This is stark contrast to those in Group A who had similar previous practices and continued to not accept the vaccine. The same pattern is true for Group B patients in comparison to Group D. Group B patients were overwhelmingly strong believers in flu shots (83.8%); however, they showed variance in the other answers. Most prominent was the fact that 44.5% of patients answered agree or strongly agree with concern about the flu shot harming the baby than concern for getting the flu, in comparison to Group D where only 17.3% agreed.

These four groups were demographically different based on an exploratory analysis (see Table 5). Group A was more likely to be non-White, particularly Hispanic/Latino and Black/African American, younger, and less educated. Group C had the highest percentage of White patients, was older, and had a higher education. Group B was more likely to be Asian, Native Hawaiian, or Pacific Islander and also had a high school degree or higher. Group D also had higher percentages of Hispanic/Latino patients, were older, and had higher education. Most significantly, and similar to our statistical analysis findings, Groups C and D who accepted the vaccine had much higher rates of receiving the educational materials and medical providers' recommendations compared to Groups A and B (Table 5).

### 3.3. Discrepancies between Patients and Medical Providers

There were some discrepancies between patient and medical provider self-reports of practices and opinions. Medical providers were asked why they believe patients do not accept the influenza vaccination. Nearly all (85.5%) chose “(patients) are afraid it is not safe,” and 65.8% chose “(patients) do not think they need vaccines.” Another third wrote in additional reasons, the majority of which were fear of needles and fear of getting sick. Medical providers' beliefs were fairly consistent with their patients'; however the majority of patients who gave answers for not accepting the vaccination were afraid of getting sick. A large amount of patients also stated that they do not think they need vaccines, they do not believe in the vaccine, or they are concerned it is not safe for their babies.

81.5% of medical providers stated they provided educational materials to their patients (53.9% always and 27.6% sometimes), but only 66.4% of patients reported receiving educational materials. There were significant differences among sites regarding educational materials. A larger number of patients at Sites 1 and 4 reported receiving educational materials, 74.3% and 68.4%, respectively, versus 60.2% in Site 2, and 60.6% at Site 3 (*p* < .01). Providers also differed within sites; 10 of the 11 providers in Site 1 reported providing educational materials always. The other 3 sites were fairly similar; more than 75% reported always or sometimes providing educational materials. Gender was also significant within sites; at Site 2 men were more likely to offer educational materials, but, at Site 3, women were more likely to offer educational materials. Similarly, 85.6% of patients reported receiving a recommendation from their medical provider while 100% of medical providers stated they recommend the vaccination. While the sites did not vary dramatically, Site 1 again was consistent in having 100% of providers recommend the vaccine at any trimester, compared to the other three sites, in which about 90% of providers recommended the vaccine at any trimester.

### 3.4. Discrepancies between Self-Reported Data and Medical Record Data

Overall, medical record data showed that only 36.1% of patients accepted the influenza vaccine. Averaging by site, 48.1% of patients accepted the influenza vaccine (Site 1: 53.6%; Site 2: 67.6%; Site 3: 38.9%; and Site 4: 32.1%). This was significantly lower than patient self-reports (77.9%). However, if sites are split between those who used prospective data (1 and 2) and those who conducted a retrospective data pull (3 and 4), medical records show that prospective data observed that 58% of patients accept the vaccine versus 80.6% of self-reports; and retrospective data observed that only 32.9% of patients accept the vaccine versus 72.8% of self-reports.

Of the patients who accepted the influenza vaccine, 74.5% accepted the influenza vaccination within their respective site's office/hospital which was similar to self-reported data. Other locations included the patients' place of employment, a referring doctor, or a commercial pharmacy. The percentage of patients who accepted the vaccine was pretty evenly split among trimesters: 30.1% during the 1st, 31.9% during the 2nd, and 38% during the 3rd. This split however may be accounted for by the patient's pregnancy dates and correspondence with flu season. We did not ask patients when they became pregnant; thus we cannot compare if the trimester of vaccination was correlated with how far long they were in their pregnancy. However, the majority of patients who were still pregnant at the end of the study accepted the influenza vaccine during the first trimester (47.4%) and 16.4% in the 2nd and 36.2% in the 3rd trimester. Patients who delivered full term accepted the influenza vaccine mainly in the 2nd trimester (43.3%) or 3rd trimester (41.9%). Similarly, 45.6% of patients who delivered prematurely accepted the vaccine in the 2nd trimester and 27.5% in the 3rd and 26.9% in the 1st trimester. The majority of patients who accepted the influenza vaccine delivered full term (47.3%) or were still pregnant (41.4%); 5.8% had a preterm delivery, and 1.4% miscarried or had a stillborn delivery.

## 4. Discussion

77.9% of patients self-reported accepting the influenza vaccination, and medical record data observed 36.1% of patients accepting the vaccination. There are a number of possibilities for the gap that exists between self-reported and medical record data, including overreporting by patients. Given that patients filled out the survey in their doctor's office, where influenza vaccines are socially desirable, patients may have overreported their willingness to accept the vaccine, when, in reality, they did not. Similarly, because the survey was voluntary, it is possible that those who filled out the questionnaire were more inclined than other patients who did not respond to the survey in favor of influenza vaccinations. The demographics of the sites which were primarily persons being White and highly educated may also skew the results and be missing a population of women who have lower rates of vaccination. Additionally, patient responses are limited by the date they filled out the survey (beginning and end of the flu season) and when their pregnancy began. It is possible, for example, that a woman received the influenza vaccine in October prior to becoming pregnant in January, and she still reported receiving the influenza vaccination. However, the majority of patients had already accepted the vaccine; only 6.8% reported a future intention.

An alternative is that medical record data was inaccurate. Two sites used data sheets over the course of the data collection period to record rates, and two sites conducted a data pull from their EMR which may be a limitation given medical provider recall error or show limitations with EMR data. Additionally, the stage of pregnancy each patient was in was difficult to compare. In one piece of EMR data, patients who delivered at the beginning of collection (e.g., September) and who had just begun their pregnancy at the end (e.g., April) were excluded given the likelihood that they would accept the vaccine during a different influenza season; however, we were not able to exclude these patients from the remaining sites. The difference between the prospective and retrospective data also suggests that EMR data may not be as accurate as intended. Retrospective studies utilizing medical records will search for items (i.e., the flu immunization) by codes primarily used for billing and are subject to errors of omission and commission by medical coders. A potential for underascertainment and misclassification bias exist. It is likely that a combination of EMR and self-reported limitation created the large gap. An additional sampling bias among patients, medical providers, and sites is also a limitation. The number of medical providers and patients varied by site which may suggest differences in practices depending on size or type of medical site.

Not surprisingly, a patient's beliefs had a strong impact on accepting the influenza vaccination while pregnant, and the greatest impact on vaccination was a patient's past immunization record (accepting the vaccine or not the same as previous years). In line with previous research, fear of contracting influenza, fear that the vaccine was unsafe, and belief of not needing the vaccine were all reasons women did not accept the vaccine, and belief of the positive benefits of vaccine was reason to accept the vaccine [8]. Our results also showed that receiving a recommendation from a doctor and receiving educational materials were significant factors in increasing the acceptance of the vaccination or impacting their beliefs, which are also in line with previous studies [8]. However, it is also important to note that only 18 patients changed their previous practices of vaccine acceptance to nonacceptance while pregnant. While the small number is better in terms of vaccine uptake, it also limits our ability to conduct further statistical analysis on why these patients changed.

The large number of patients who changed behaviors from nonacceptance to acceptance of vaccine indicates a success in increasing immunization among pregnant women. The largest contrast within these patients compared to those who did not change their behavior was concern for the baby. Future research should consider ways to more effectively communicate to patients the safety and importance of the vaccine for the health of the baby, as well as research the difference between educational levels. Patients with a high school degree, but not more than a high school degree, were more likely to get the influenza vaccine and have positive beliefs about the vaccine, which could suggest targeting educational materials towards patients with lower and higher educational levels. While it seems contradictory that more education would reduce influenza vaccinations, one hypothesis could be that more educated women research a variety of media sources, including antivaccine reports and blogs. While not statistically significant in our logistic regression analyses, our exploratory analysis of patients who changed behaviors suggests that non-White women are less likely to accept the vaccine. These results are similar to other studies, which have shown that non-White and lower educated women are less likely to accept the flu vaccine as well [8]. However, our sample was primarily White and highly educated and therefore these results cannot be generalized to the larger US population without additional research and analysis.

## 5. Conclusion

In summary, our findings showed the importance of providing educational materials and a recommendation from a medical provider in acceptance of influenza vaccination. In addition, our findings suggested that non-White and less educated women may be less likely to accept the vaccine while pregnant. Future research should explore reasons pregnant patients would be inclined to change previous immunization behavior more thoroughly. In addition, our findings showed discrepancies between patients and medical providers self-reports and medical record data. Improving medical record tools, communication between medical providers and patients, and determining more accurate ways of measuring self-reported vaccination rates should also be considered for future research.

## Supplementary Material

Supplementary material includes two tables; results from the binary logistic regression and results from the multinomial logistic regression. The binary logistic regression assessed a patient's acceptance of the influenza vaccine (yes or no) using a number of independent variables such as demographic information and interactions with physicians (receiving a medical provider's recommendation and receiving educational materials). The multinomial logistic regression assessed patient beliefs towards the influenza vaccine. Scores from 4 questions were grouped into three categories (4-9, 10-15, 16-20; the higher the more positive belief) for the analysis. Independent variables were also demographic information and interactions with physicians.

## Figures and Tables

**Table 1:** Demographic information.

Data collection, flu season	Site 1	Site 2	Site 3	Site 4	Total
	2013-2014	2013-2014	2014-2015	2014-2015^*∗∗*^	—
*Medical provider demographics*					

Sample size	11	33	8	24	76
Gender					
* Female*	54.5%	72.7%	62.5%	79.2%	71.1%
* Male*	45.5%	27.3%	37.5%	20.8%	28.9%
Mean year of birth^*∗*^					
* Female*	1971	1978	1966	1977	1976
* Male*	1965	1960	1961	1966	1962
* Total*	1968	1973	1965	1975	1972
Years in practice					
* Female*	13.7	5.25	14.2	9.6	8.5
* Male*	17.2	23.6	20	15	19.7
* Total*	15.27	10.24	16.38	10.78	11.8

*Patient demographics*					

Sample size	280	365	66	273	984
Race					
* Hispanic, Latino, or Spanish*	34.9%	11.2%	6.9%	1.5%	13.1%
* White*	46%	66.8%	40.3%	96.7%	59%
* Black or African American*	25.2%	17.3%	41.7%	1.5%	14.8%
* American Indian or Alaska Native *	1.4%	0.3%	—	0.4%	.05%
* Asian, Native Hawaiian, or other Pacific Islander*	12.9%	7.4%	9.7%	1.8%	7.7%
* Other*	12.9%	6.6%	1.4%	1.8%	5.9%
Age					
* Mean*	30.86	28.97	30.98	28.69	29.6
* 18–27*	23.1%	41.2%	19.7%	42.4%	35%
* 28–34*	55.2%	41.8%	54.5%	45%	47.3%
* 35–47*	21.7%	17%	25.8%	12.5%	17.7%
Education					
* Less than high school diploma*	2.5%	9.6%	—	5.2%	5.7%
* High school diploma, GED, or equivalent*	12.2%	28.7%	13.6%	27.4%	22.5%
* More than high school diploma*	85.3%	61.7%	86.4%	67.4%	71.5%

^*∗*^Statistically significant at the 95% level.

^*∗∗*^Medical record data also collected for the 2013-2014 flu season.

**Table 2:** Physician beliefs.

	Site 1 (%)	Site 2 (%)	Site 3 (%)	Site 4 (%)	Total (%)		
					F	M	T
Safety of administration in 1st trimester							
* Not concerned*	63.6	57.6	87.5	29.2	48.1	63.6	52.6
* Slightly concerned*	—	12.1	—	33.3	16.7	13.6	15.8
* Concerned*	18.2	9.1	—	16.7	14.8	4.5	11.8
* Very concerned*	18.2	21.2	12.5	20.8	20.4	18.2	19.7

Health of mother and fetus if flu is contracted							
* Not concerned*	—	—	—	—	—	—	—
* Slightly concerned*	—	3	—	—	1.9	—	1.3
* Concerned*	18.2	6.1	12.5	29.2	18.5	9.1	15.8
* Very concerned*	81.8	90.9	87.5	70.8	79.6	90.9	82.9

**Table 3:** Physician results.

	Provide educational materials			Recommend vaccine	
	*Never*	*Sometimes*	*Always*	*Any trimester*	*Any time after 1st trimester*
*Site*					
Site 1	—	9.1%	90.9%	100%	—
Site 2	21.2%	21.2%	51.5%	87.9%	12.1%
Site 3	25%	25%	50%	87.5%	12.5%
Site 4	12.5%	45.8%	41.7%	91.3%	8.7%

*Total*	*15.8%*	*27.6%*	*53.9%*	*90.7%*	*9.3%*

**Table 4:** Group characteristics.

	Group A *N* = 168	Group B *N* = 18	Group C *N* = 167	Group D *N* = 489	Pearson Chi-square *p* value
*Influenza vaccine practices*					
Accepted vaccine in previous years	No	Yes	No	Yes	
Accepted vaccine while pregnant	No	No	Yes	Yes	

*Race*					
Hispanic, Latino, or Spanish	18.6%	5.6%	7.8%	16.8%	.001
White	61.7%	66.7%	70.1%	69.7%	
Black or African American	19.8%	11.1%	19.8%	15%	
American Indian or Alaska Native	2.4%	—	0.6%	—	
Asian, Native Hawaiian, or other Pacific Islander	9%	22.2%	3.6%	6.8%	
Other	6.6%	—	6%	6.8%	

*Age*					
18–27	49.7%	35.3%	32.9%	30.7%	.000
28–34	36.5%	64.7%	52.7%	48.7%	
35–47	13.8%	—	14.4%	20.6%	

*Education*					
Less than high school diploma	3.6%	—	6%	4.8%	.000
High school diploma, GED, or equivalent	37.5%	33.3%	23.4%	17.4%	
More than high school diploma	58.9%	66.7%	70.7%	77.9%	

*Received educational materials*	50.9%	44.4%	70.7%	71.8%	.000

*Received medical provider's recommendation*	73.5%	61.1%	91.6%	89%	.000

**Table 5:** Patient flu vaccine practices and beliefs.

	Opinion (%)				
	*Strongly disagree*	*Disagree*	*Don't know*	*Agree*	*Strongly agree*
I am a strong believer that people should get flu shots					
* Group A*	24.6	26.4	46.1	2.4	0.6
* Group B*	5.6	—	27.8	44.4	22.2
* Group C*	6.7	20.6	50.3	18.2	4.2
* Group D*	—	0.4	15.8	40.5	43.3

It is important for my health and safety to get a flu shot while I am pregnant					
* Group A*	25.1	34.7	34.1	6	—
* Group B*	5.6	—	27.8	44.4	22.2
* Group C*	1.8	4.8	24.8	40.6	27.9
* Group D*	—	0.2	6.3	33.9	59.5

It is important for the health and safety of my baby that I get a flu shot while I am pregnant					
* Group A*	4.8	16.4	26.1	28.5	24.2
* Group B*	5.6	5.6	33.3	33.3	22.2
* Group C*	0.6	4.9	22	42.1	30.5
* Group D*	—	0.4	8	31.8	59.8

I am more worried about the flu shot harming my baby than I am about what would happen if I get the flu					
* Group A*	4.8	16.4	26.1	28.5	24.2
* Group B*	—	27.8	27.8	5.6	38.9
* Group C*	12.3	38.9	27.2	15.4	6.2
* Group D*	27.3	40.9	14.6	7.6	9.7

Mean score					
* Group A*	9.24				
* Group B*	13.61				
* Group C*	14.11				
* Group D*	16.99				
