# Supplementary material for: Influenza Vaccination among Pregnant Women: Patient Beliefs and Medical Provider Practices
Source: Infect Dis Obstet Gynecol. 2016 Jul 31;2016:3281975. doi: 10.1155/2016/3281975 (PMC4983379; doi:10.1155/2016/3281975)
Supplement: Supplementary file 1 — Supplementary material includes two tables; results from the binary logistic regression and results from the multinomial logistic regression. The binary logistic regression assessed a patient's acceptance of the influenza vaccine (yes or no) using a number of independent variables such as demographic information and interactions with physicians (receiving a medical provider's recommendation and receiving educational materials). The multinomial logistic regression assessed patient beliefs towards the influenza vaccine. Scores from 4 questions were grouped into three categories (4-9, 10-15, 16-20; the higher the more positive belief) for the analysis. Independent variables were also demographic information and interactions with physicians. [file 3281975.f1.pdf]

Supplementary Tables

Table 6: Binary Logistic Regression Results

Independent Variables	Exp (B)	P value
Site 1	1.249	.531
Site 2	.561	.270
Site 3	.919	.837
High school degree	1.282	.645
More than a high school degree	1.983	.194
Age	.994	.800
Medical Provider's Recommendation	2.603	.004*
Educational materials	1.400	.207
Flu Vaccine Belief Score	1.708	.000*
Latino	3.269	.186
Black/African American	1.292	.497
Asian	31783249.210	1.000
American Indian	.599	.330
Other	.401	.381
Multiple Races	1.875	.151
Flu shot previous years	4.867	.000*
Sometimes received flu shot in previous years	1.389	.497
Constant	.000	

* Statistically significant at the 95% level

Table 7: Multinomial Logistic Regression Results

Independent Variables		Exp (B)	P value
Group 2 – Scores 10-15	Site 1	.682	.358
	Site 2	.524	.209
	Site 3	.645	.241
	High school degree	.137	.057
	More than a high school degree	.154	.074
	Age	1.056	.027*
	Medical Provider's Recommendation	.779	.431
	Educational materials	1.542	.096
	Latino	2.558	.383
	Black/African American	1.231	.578
	American Indian	2.568	.220
	Other	.487	.365
	Multiple Races	1.285	.561
Group 3 – Scores 16-20	Site 1	.573	.178
	Site 2	.460	.131
	Site 3	.564	.124
	High school degree	.015	.079
	More than a high school degree	.059	.138
	Age	1.095	.000*
	Medical Provider's Recommendation	1.871	.069
	Educational materials	2.287	.002*
	Latino	1.455	.733
	Black/African American	.897	.774
	American Indian	3.296	.119
	Other	.832	.808
	Multiple Races	1.271	.577

* Statistically significant at the 95% level
